# Supplementary material for: Antibody escape by polyomavirus capsid mutation facilitates neurovirulence
Source: eLife. 2020 Sep 17;9:e61056. doi: 10.7554/eLife.61056 (PMC7541085; doi:10.7554/eLife.61056)
Supplement: Supplementary file 1. — Independent datasets were collected for MuPyV and the MuPyV-Fab complex. [file elife-61056-supp1.docx]

|  | MuPyV | MuPyV-Fab |
| --- | --- | --- |
| Microscope/Detector | Titan Krios |  |
| Detector | Falcon 3 |  |
| Magnification | x59,000 |  |
| Voltage (kV) | 300 |  |
| Electron exposure | 45 |  |
| Defocus range (µm) | 1. – 3.0 |  |
| Pixel Size | 1.1 Å |  |
| Micrographs (total) | 1,756 | 1,811 |
| Micrographs (used) | 1,750 | 1,804 |
| Particles (total) | 17,416 | 9,811 |
| Particles (used) | 15,499 | 9,146 |
