# Supplementary material for: Antibody escape by polyomavirus capsid mutation facilitates neurovirulence
Source: eLife. 2020 Sep 17;9:e61056. doi: 10.7554/eLife.61056 (PMC7541085; doi:10.7554/eLife.61056)
Supplement: Supplementary file 2. — Local refinement allowed models to be built into the higher resolution capsomer maps. [file elife-61056-supp2.docx]

| Sample | MuPyV |  |  | MuPyV-Fab |  |  |
| --- | --- | --- | --- | --- | --- | --- |
| Map composition | capsid | capsomer (pentavalent) | capsomer (hexavalent) | capsid | capsomer (pentavalent) | capsomer (hexavalent) |
| Particle number | 15,499 | 185,988 | 929,940 | 9,146 | 109,752 | 548,769 |
| Symmetry imposed | I1 | C5 | C1 | I1 | C5 | C1 |
| Map resolution (Å) | 3.9Å | 2.9 | 2.9 | 4.2Å | 3.2 | 3.3 |
| Local resolution (Å) |  | 2.7 – 3.1 | 2.7 – 3.1 |  | 2.9 – 3.8 | 3.0 – 3.8 |
| Model composition |  | VP1 | VP1 |  | VP1, 8A7H5 Fab | VP1, 8A7H5 Fab |
|  |  |  |  |  |  |  |
| VP1 |  | chain F (x5) | chain A-E |  | chain F (x5) | chain A-E |
| Protein residues |  | 1660 | 1687 |  | 1660 | 1687 |
| RMS bonds (Å) |  | 0.0044 | 0.0043 |  | 0.0073 | 0.0078 |
| RMS angles (°) |  | 0.76 | 0.72 |  | 0.82 | 0.84 |
| Validation |  |  |  |  |  |  |
| Molprobity Score |  | 2.97 | 2.67 |  | 2.86 | 2.82 |
| Clashscore |  | 11.99 | 9.16 |  | 11.59 | 9.98 |
| Rotamer outliers (%) |  | 11.60 | 8.09 |  | 9.29 | 9.10 |
| Ramachandran plot |  |  |  |  |  |  |
| Favored (%) |  | 89.43 | 91.91 |  | 90.06 | 89.33 |
| Outliers (%) |  | 0.29 | 0.39 |  | 0.29 | 0.34 |
|  |  |  |  |  |  |  |
| Fab |  |  |  |  | 1x | 5x |
| Protein residues |  |  |  |  | 215 | 1070 |
| RMS bonds (Å) |  |  |  |  | 0.0043 | 0.0048 |
| RMS angles (°) |  |  |  |  | 0.74 | 0.85 |
| Validation |  |  |  |  |  |  |
| Molprobity Score |  |  |  |  | 2.95 | 3.35 |
| Clashscore |  |  |  |  | 18.74 | 16.71 |
| Rotamer outliers (%) |  |  |  |  | 8.33 | 19.27 |
| Ramachandran plot |  |  |  |  |  |  |
| Favored (%) |  |  |  |  | 92.31 | 85.78 |
| Outliers (%) |  |  |  |  | 0.00 | 0.46 |
