# Supplementary material for: Antibody escape by polyomavirus capsid mutation facilitates neurovirulence
Source: eLife. 2020 Sep 17;9:e61056. doi: 10.7554/eLife.61056 (PMC7541085; doi:10.7554/eLife.61056)
Supplement: Supplementary file 3. — The majority of Fab contacts are through the heavy chain, with minor contributions from the light chain. [file elife-61056-supp3.docx]

| CDR |  |  | **VP1 residue** |
| --- | --- | --- | --- |
| L1 | Tyr | 30 | 141, 292 |
| L3 | Asn | 92 | 83 |
|  | Ala | 93 | 83 |
| H1 | Ser | 26 | 67, 68 |
| H2 | Ser | 49 | 77 |
|  | Ala | 50 | 77 |
|  | Asp | 52 | 91 |
|  | Asn | 69 | 68 |
| H3 | Tyr | 96 | 293 |
|  | Thr | 98 | 293 |
|  | Asp | 99 | 77, 78, 80 |
|  | His | 100 | 80, 151, 294, 296 |
|  | Phe | 101 | 80 |
|  | Tyr | 102 | 83 |
|  | Asp | 103 | 294 |
|  | Trp | 104 | 294 |
