# Supplementary material for: Antibody escape by polyomavirus capsid mutation facilitates neurovirulence
Source: eLife. 2020 Sep 17;9:e61056. doi: 10.7554/eLife.61056 (PMC7541085; doi:10.7554/eLife.61056)
Supplement: Supplementary file 4. — Capsomer-based local refinement allowed rapid refinement of polyomavirus to high resolution, using only a modest particle number. [file elife-61056-supp4.docx]

| **Virus** | Avian polyomavirus | BK polyomavirus | BK polyomavirus | BK polyomavirus | Murine polyomavirus | Murine polyomavirus |
| --- | --- | --- | --- | --- | --- | --- |
| **Author** | Shen et al. | Hurdiss et al. | Lindner et al. | Hurdiss et al. | This study | This study |
| **PMID** | 21239031 | 26996963 | 30824324 | 29706532 | -- | -- |
| **PDB** | 3IYS | 5FUA | 6GG0 | 6ESB | -- | -- |
| **Year** | 2011 | 2016 | 2019 | 2018 | 2020 | 2020 |
| **Ligand** | N/A | N/A | scFv | oligosacch. | Fab | none |
| **Resolution** | 11.3Å | 7.6Å | 4.2Å | 3.4Å | 3.2Å [2.9 – 3.8Å] | 2.9Å [2.7 – 3.1Å] |
| **Particles** | 5,338 | 2,237 | 5,000 | 40,334 | 9,171 | 15,499 |
| **Pixel Size** | ?? | 1.9Å | 1.1Å | 1.1Å | 1.1Å | 1.1Å |
| **Microscope** | Tecnai-F30 | Tecnai-F20 | Krios | Krios | Krios | Krios |
| **Detector** | film | K2 | K2 | Falcon III | Falcon III | Falcon III |
