# Supplementary material for: Antibody escape by polyomavirus capsid mutation facilitates neurovirulence
Source: eLife. 2020 Sep 17;9:e61056. doi: 10.7554/eLife.61056 (PMC7541085; doi:10.7554/eLife.61056)
Supplement: Supplementary file 6. — Sequences of oligonucleotides used for site-directed mutagenesis, qPCR, sequencing. [file elife-61056-supp6.docx]

**Oligonucleotides**

| Designation | Reference | Sequence 5’-3’ |
| --- | --- | --- |
| V296F Mutagenesis Forward | This paper | CTCTCCAGTGATGGAAATCATAGTTTCTTG |
| V296F Mutagenesis Reverse | This paper | CAAGAAACTATGATTTCCATCACTGGAGAG |
| V296A Mutagenesis Forward | This paper | GCCCTCTCCAGTGATGGGCATCATAGTTTCTTGTAACTC |
| V296A Mutagenesis Reverse | This paper | GAGTTACAAGAAACTATGATGCCCATCACTGGAGAGGGC |
| V296Y Mutagenesis Forward | This paper | GGGAAGCCCTCTCCAGTGATGGTAATCATAGTTTCTTGTAACTC |
| V296Y Mutagenesis Reverse | This paper | GAGTTACAAGAAACTATGATTACCATCACTGGAGAGGGCTTCCC |
| V296I Mutagenesis Forward | This paper | GGGAAGCCCTCTCCAGTGATGGATATCATAGTTTCTTGTAACTC |
| V296I Mutagenesis Reverse | This paper | GAGTTACAAGAAACTATGATATCCATCACTGGAGAGGGCTTCCC |
| V296W Mutagenesis Forward | This paper | GGGAAGCCCTCTCCAGTGATGCCAATCATAGTTTCTTGTAACTC |
| V296W Mutagenesis Reverse | This paper | GAGTTACAAGAAACTATGATTGGCATCACTGGAGAGGGCTTCCC |
| N293F Mutagenesis Forward | This paper | GTGATGGACATCATAGAATCTTGTAACTCTCCAGCCCATTATATC |
| N293F Mutagenesis Reverse | This paper | GATATAATGGGCTGGAGAGTTACAAGATTCTATGATGTCCATCAC |
| N293Y Mutagenesis Forward | This paper | GTGATGGACATCATAGTATCTTGTAACTCTCCAGCCCATTATATC |
| N293Y Mutagenesis Reverse | This paper | GATATAATGGGCTGGAGAGTTACAAGATACTATGATGTCCATCAC |
| T291D Mutagenesis Forward | This paper | GTGATGGACATCATAGTTTCTGTCAACTCTCCAGCCCATTATATC |
| T291D Mutagenesis Reverse | This paper | GATATAATGGGCTGGAGAGTTGACAGAAACTATGATGTCCATCAC |
| H139R Mutagenesis Forward | This paper | CTGTGGGTTTGTTGAACCCACGCACATCTAACAGTGAGCCAGAGC |
| H139R Mutagenesis Reverse | This paper | GCTCTGGCTCACTGTTAGATGTGCGTGGGTTCAACAAACCCACAG |
| R77N Mutagenesis Forward | This paper | GTAGCCAAATTAATCCCATTGCTCCAACCATAGTATTGCCCTCCC |
| R77N Mutagenesis Reverse | This paper | GGGAGGGCAATACTATGGTTGGAGCAATGGGATTAATTTGGCTAC |
| N80K Mutagenesis Forward | This paper | CTATGGTTGGAGCAGAGGGATTAAGTTGGCTACATCAGATACAGAGGATTCCCC |
| N80K Mutagenesis Reverse | This paper | GGGGAATCCTCTGTATCTGATGTAGCCAACTTAATCCCTCTGCTCCAACCATAG |
| ∆294 Mutagenesis Forward | This paper | CTGGGAAGCCCTCTCCAGTGATGGACATCATTTCTTGTAACTCTCCAGCCCATTATATC |
| ∆294 Mutagenesis Reverse | This paper | GATATAATGGGCTGGAGAGTTACAAGAAATGATGTCCATCACTGGAGAGGGCTTCCCAG |
| LT DNA qPCR Forward | Wilson et al., 2012 | CGCACATACTGCTGGAAGAAGA |
| LT DNA qPCR Reverse | Wilson et al., 2012 | TCTTGGTCGCTTTCTGGATACAG |
| LT DNA qPCR probe | Wilson et al., 2012 | ATCCTTGTGTTGCTGAGCCCGATG |
| V296F DNA qPCR Detection Forward | This paper | CCCTCTCCAGTGATGGAA |
| V296F DNA qPCR Detection Reverse | This paper | AGAACACAAGGTACTTTGGC |
| LT mRNA qPCR Forward | Maru et al., 2017 | AGGAATTGAACAGTCTCTGGG |
| LT mRNA qPCR Reverse | Maru et al., 2017 | GTCATCGTGTAGTGGACTGTG |
| LT mRNA qPCR probe | Maru et al., 2017 | AACCGGCTTCCAGGGCTCT |
| VP1 Amplification and Sequencing Forward | This paper | 5CGACCCCTTGAAGGACATATGTGAA |
| VP1 Amplification and Sequencing Reverse | This paper | CACCTACTTGGGCAACAGTCA |
